# Aberrant Short Tandem Repeats: Pathogenicity, Mechanisms, Detection, and Roles in Neuropsychiatric Disorders

**DOI:** 10.3390/genes16040406

**Published:** 2025-03-30

**Authors:** Yuzhong Liu, Kun Xia

**Affiliations:** 1 Institute of Cytology and Genetics, School of Basic Medical Sciences, Hengyang Medical School, University of South China, Hengyang 421001, China; liuyuzhong25@163.com; 2 MOE Key Lab of Rare Pediatric Diseases, School of Basic Medicine, Hengyang Medical College, University of South China, Hengyang 421001, China

**Keywords:** STRs, neuropsychiatric disorders, repeat expansions, genetic mechanisms, sequencing technologies, autism, schizophrenia

## Abstract

Short tandem repeat (STR) sequences are highly variable DNA segments that significantly contribute to human neurodegenerative disorders, highlighting their crucial role in neuropsychiatric conditions. This article examines the pathogenicity of abnormal STRs and classifies tandem repeat expansion disorders(TREDs), emphasizing their genetic characteristics, mechanisms of action, detection methods, and associated animal models. STR expansions exhibit complex genetic patterns that affect the age of onset and symptom severity. These expansions disrupt gene function through mechanisms such as gene silencing, toxic gain-of-function mutations leading to RNA and protein toxicity, and the generation of toxic peptides via repeat-associated non-AUG (RAN) translation. Advances in sequencing technologies—from traditional PCR and Southern blotting to next-generation and long-read sequencing—have enhanced the accuracy of STR variation detection. Research utilizing these technologies has linked STR expansions to a range of neuropsychiatric disorders, including autism spectrum disorders and schizophrenia, highlighting their contribution to disease risk and phenotypic expression through effects on genes involved in neurodevelopment, synaptic function, and neuronal signaling. Therefore, further investigation is essential to elucidate the intricate interplay between STRs and neuropsychiatric diseases, paving the way for improved diagnostic and therapeutic strategies.

## 1. Introduction of Short Tandem Repeats and Tandem Repeat Expansion Disorders

Short tandem repeats (STRs) are tandemly repeated DNA sequences consisting of repeat motifs of 1–6 base pairs (bp) [1,2]. STRs represent one of the most variable types of DNA sequences within the genome, constituting approximately 3–5% of the human genome—exceeding the proportion occupied by protein-coding genes [1,2,3]. These sequences are ubiquitously present across nearly all human chromosomes, yet their distribution is notably uneven [4,5,6]. Chromosome 19 boasts the highest density of STRs [4], whereas chromosomes 1 and X exhibit relatively lower STR densities [4,5,6]. The majority of STRs are located in non-coding regions, such as promoters, enhancers, and introns [4], where they regulate gene expression by influencing DNA methylation, chromatin structure, and RNA processing [7,8]. In contrast, the minority of STRs situated in coding regions are subject to stronger selective pressure due to their potential impact on protein function and structure [9,10,11]. Non-coding STRs are primarily associated with the regulation of genetic variation [12,13], particularly regarding modifiers in conditions such as Huntington’s disease (HD). Beyond the CAG expansion within the *HTT* gene, several other STRs—such as those found within the introns of the *MLH1* and *MSH3* genes—modulate disease onset and severity by influencing DNA repair efficiency [14,15]. Fuchs endothelial corneal dystrophy (FECD), the non-coding CTG repeat expansion in the *TCF4* gene, causes RNA aggregation, triggering cell death in corneal endothelial cells through the activation of the unfolded protein response [16]. On the other hand, coding STRs may exert direct toxic effects on proteins [12,13]. For example, spinal bulbar muscular atrophy (SBMA) is closely linked to the expansion of CAG trinucleotide repeats within the androgen receptor (*AR*) gene [14]. When the number of CAG repeats exceeds the normal threshold (typically over 38 repeats), the resulting polyglutamine (polyQ) protein can form misfolded aggregates, which in turn leads to neurotoxicity [17,18].

Tandem Repeat Expansion Disorders (TREDs) are a class of hereditary diseases caused by the abnormal expansion of specific DNA sequence repeats beyond their physiological range [19,20,21]. These repeat expansions typically occur in either coding or non-coding regions of the genome, resulting in altered protein function or abnormal gene expression [21]. The etiology of these diseases may involve genetic factors, environmental influences, or a complex interplay between both [22,23]. In recent years, with the advancements in sequencing technologies such as targeted sequencing and whole-genome long-read sequencing (LRS), substantial progress has been made in identifying new repeat expansion loci and elucidating motif structures, interruption patterns, and allelic diversity [24,25,26]. Increasing evidence suggests that TREDs predominantly affect the nervous system [27,28,29,30]. To date, STR expansions have been implicated in 65 neurological diseases and 14 neuromuscular diseases [31]. Based on the systems they affect, repeat expansion disorders can be broadly categorized into Neurological Disorders, Psychiatric/Mental Disorders (Table 1) and Non-Neuropsychiatric Disorders [32,33]. Neurological Disorders associated with tandem repeat expansions (TREs) are characterized by degenerative changes in the central or peripheral nervous system. These disorders involve various types of repeats and pathogenic mechanisms. Examples include Fragile X Syndrome (FXS) [34,35,36], HD [10,37,38], and Neuronal Intranuclear Inclusion Disease (NIID) [39]. In these conditions, abnormal STR expansions directly result in gene function loss or toxic protein accumulation, leading to neurological dysfunction [40,41,42,43,44]. Psychiatric or Mental Disorders within the spectrum of repeat expansion disorders are characterized by psychiatric symptoms caused directly or indirectly by abnormal TREs in specific genes. The pathological mechanisms underlying these disorders are complex, such as RNA toxicity or protein aggregation [18] and epigenetic regulation or synaptic plasticity alterations [18,45,46,47,48]. Examples include Autism Spectrum Disorder (ASD) [28,29], Fragile X-associated Neuropsychiatric Disorders (FXAND), Bipolar Disorder (BD), and Schizophrenia (SCZ) [30,49]. In these cases, STR variations increase susceptibility by modulating the expression and function of multiple related genes [50,51,52,53]. Non-Neuropsychiatric Disorders are repeat expansion disorders that do not directly involve the central nervous system or mental/behavioral functions [54]. Examples include Blepharophimosis, Ptosis, and Epicanthus Inversus Syndrome (BPES) [55], Congenital Central Hypoventilation Syndrome (CCHS) [56], and Cleidocranial Dysplasia (CLCD) [57]. The pathological manifestations of these disorders primarily impact muscles, the metabolic system, epithelial tissues, the cardiovascular system, or the endocrine organs, demonstrating the broad systemic pathogenic effects of repeat expansions across multiple organ systems [55,56,57]. In-depth research into STRs not only enhances our understanding of the pathogenic mechanisms underlying these disorders but also provides new avenues for their diagnosis and treatment.

This review provides an overview of the genetic patterns and characteristics of diseases associated with STRs and highlights the significant role of STR expansions in various genetic and neurodevelopmental disorders, such as HD, muscular dystrophy, and ASD. Additionally, the review explores the mechanisms by which STR expansions lead to disease, including RNA and protein toxicity, RAN translation, and gene silencing. This review particularly focuses on the role of STRs in neuropsychiatric disorders and discusses the advancements in sequencing technologies and their application in research within this field.

## 2. Methods for Detecting Variations in STR Sequence Composition

**Figure 1:**
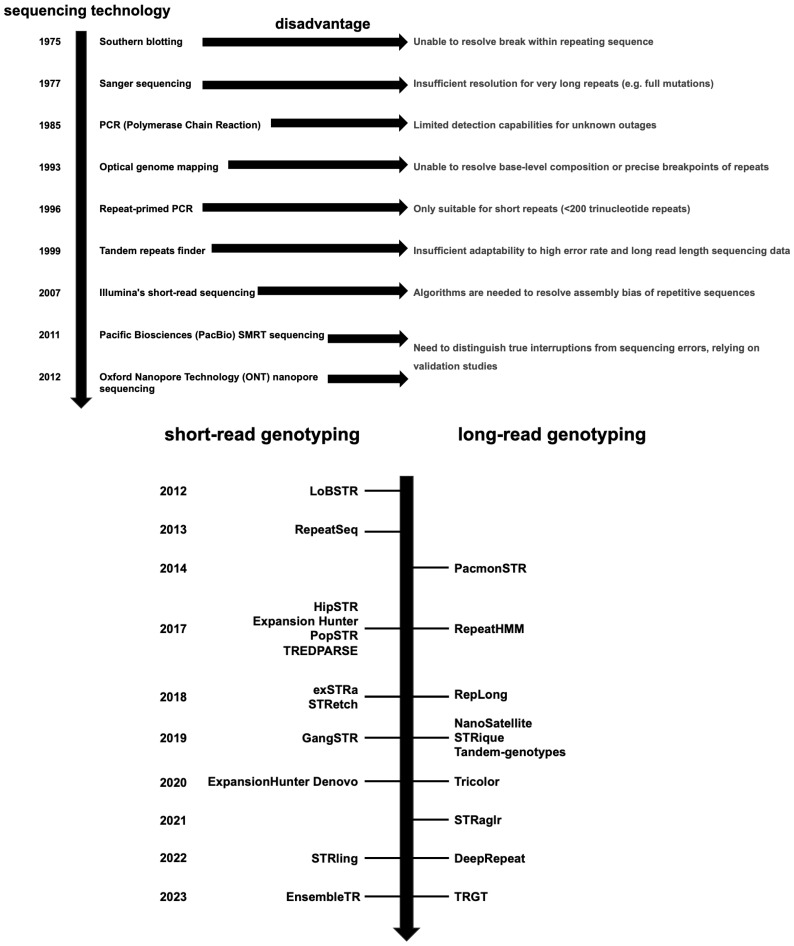
STR Identification Technology Development Timeline. LoBSTR: a tool specifically designed for early short-read genotyping; RepeatSeq: an advanced tool aimed at optimizing short-read data processing; PacmonSTR: a tool that combines short-read and long-read data to enhance analytical capabilities; HipSTR: an innovative tool for efficiently detecting tandem repeat sequences; Expansion Hunter: detects disease-related expansions of long STRs; PopSTR: conducts population-scale STR genotyping; TREDPARSE: an improved alignment algorithm; RepeatHMM: modeling using Hidden Markov Models; exSTRA: a powerful tool that demonstrates exceptional performance in handling low-coverage data; STRetch: analyzes the expansion and variation of STRs in the genome; RepLong: an optimized processing workflow; GangSTR: a tool that incorporates population genetics models to enrich the depth of analysis; NanoSatellite: designed specifically for Oxford Nanopore Technologies (ONT) data; STRique: a tool that integrates short-read and long-read data; Tandem-genotypes: specifically designed for population-scale STR genotyping; EHdn: a robust tool for de novo identification of repeat sequences; Tricolor: a technology utilizing multicolor fluorescent labeling; STRaglr: an enhanced algorithm for optimized detection; STRling: a tool that performs joint analysis of short-read and long-read data to obtain more comprehensive results; DeepRepeat: the application of deep learning techniques; EnsembleTR: a tool that significantly enhances performance through ensemble learning methods; TRGT: an advanced genomic analysis algorithm.

With technological advancements, the detection of variations in the sequence composition of STRs has undergone significant transformation(Figure 1). Initially, PCR-based methods and Southern blot analysis were the primary techniques utilized by clinical laboratories to diagnose repeat expansion disorders [58,59,60,61]. Southern blotting was considered the gold standard for genotyping highly expanded full mutations (>250 triplet repeats), although it was incapable of characterizing interruptions within the repeat sequences [19,62,63]. Conventional PCR assays, which involve amplifying the repeat region with two primers followed by restriction digestion, can distinguish interrupted alleles from uninterrupted ones, but these require prior knowledge of the interruption motif and its sequence [64,65].

However, these early methods faced limitations in detecting interruptions within repeats [31]. The advent of Sanger sequencing and improved PCR techniques significantly enhanced the ability to resolve repeat length and sequence composition [31]. Sanger sequencing allows for the estimation of repeat lengths and can determine the sequence composition of alleles with up to approximately 250–300 triplet repeats [66]. Improved PCR methods, such as repeat-primed PCR (RP-PCR), enable the mapping of sequence composition variations by generating heterogeneous mixtures of amplified fragments [62,67,68,69,70]. RP-PCR can detect most repeat expansions and reveal the positions of interruptions within repeats, but its genotyping capability is limited to repeats shorter than approximately 200 triplets [67,71].

Optical genome mapping (OGM) has emerged as another approach for identifying structural variations, including copy number variations and structural changes larger than 500 base pairs [72]. OGM provides indirect evidence of repeat expansions by identifying increased distances between tags flanking STR regions [73]. However, it cannot resolve precise breakpoints or the base-by-base composition of the repeats [74].

As technology progresses, more advanced sequencing techniques are addressing the limitations of molecular detection methods. Both short-read sequencing (NGS) and LRS have been applied to achieve higher-resolution STR analysis [31]. NGS offers a high-throughput platform for characterizing STR sequence compositions. Bioinformatics tools, such as ExpansionHunter Denovo (EHdn) [75] and STRling [76], can identify both reference and non-reference expansions. Nevertheless, resolving highly repetitive sequences and addressing technology-specific biases require specialized algorithms and validation studies [77].

LRS technologies, such as those developed by Pacific Biosciences or Oxford Nanopore Technologies, are capable of generating read lengths of approximately 15–20 kilobases or more [19,78,79,80,81,82,83,84,85,86]. This allows for the resolution of non-canonical motifs and simultaneous methylation analysis [24]. These technologies can fully span most repetitive expansions, providing detailed characterization of pathogenic repeat expansions [19,78,79,80,81,82,83,84,85,86]. A significant challenge in LRS data analysis lies in distinguishing true interruptions from sequencing errors, necessitating rigorous validation studies [80,87]. As LRS becomes more affordable and sequencing accuracy continues to improve, it may evolve into the standard method for detecting repeat expansions [88].

In addition to the continuous advancements in sequencing technologies, the genetic methods used to analyze the relationship between STRs and affected disorders have also expanded significantly. For instance, linkage analysis methods can be employed in family studies that utilize STR markers to co-segregate with disease phenotypes [89], thereby localizing pathogenic genes [90,91]. Typically, a lod score exceeding 3 indicates significant linkage [90,91,92]; however, this method tends to be less sensitive for polygenic disorders [93], such as those characterized by multiple genetic factors. To enhance accuracy, high heterozygosity STR markers and parametric models, such as the Elston–Stewart algorithm, are often utilized [94].

Moreover, Genome-Wide Association Studies (GWAS) can be employed to compare the frequency differences of STRs between case and control groups, facilitating the identification of risk loci. For example, ASD research has uncovered 54 significantly associated STRs through GWAS [95]. Additionally, expression STRs (eSTRs) analysis integrates RNA-seq data to identify STRs that regulate gene expression. These eSTRs can account for 10–15% of gene expression variance and are associated with complex diseases such as Crohn’s disease [96]. Polygenic Risk Scores (PRS) are then utilized to evaluate the cumulative effects of multiple STR expansions; for instance, patients with SCZ exhibit a higher burden of STR expansions [97].

With the development of sequencing technology, it is now possible to directly determine the relationship between repeat sequences and diseases by sequencing [83]. For example, LRS utilizing nanopore or SMRT technology allows for the direct measurement of long repeat sequences, enabling the precise analysis of pathogenic expansions, such as the CAG repeat in HD, along with their methylation status [24,98]. Targeted sequencing further enhances clinical applicability by designing probes for known pathogenic STRs (e.g., *FMR1*, *HTT*), thus reducing costs [99,100].

In addition, bioinformatics tools can be employed to identify the relationship between STRs and diseases. Tools such as LobSTR [7], HipSTR [101], and GangSTR [102] can infer STR lengths from short-read data, though challenges remain regarding alignment errors and GC content bias. Expanded detection tools like ExpansionHunter [99] and STRique [100] are designed to identify pathogenic expansions. Furthermore, three-dimensional genome analysis techniques (such as Hi-C) have revealed co-localization between STRs and chromatin domain boundaries [103]. These advancements in technology have rendered the investigation of the relationship between STRs and diseases more comprehensive and profound.

## 3. The Genetic Characteristics of Neurological Disorders Caused by Repeat Expansion Disorders

Large expansions of STRs can become pathogenic and are the underlying cause of various primary neurological disorders [19]. The earliest identified repeat expansion disorder can be traced back to FXS, which was the first repeat expansion disorder to have its molecular basis uncovered. As early as 1991, researchers employed Southern blotting along with other cytogenetic and molecular techniques to ascertain that the abnormal expansion of the CGG trinucleotide repeat sequence within the 5′ UTR of the *FMR1* gene was the causative factor of FXS. These expanded STRs lead to the silencing of *FMR1* gene transcription through promoter methylation, ultimately resulting in the silencing of the gene and subsequent neuronal dysfunction [104,105,106,107]. Since then, STR expansion disorders and the pathogenic mechanisms of STRs have been gradually identified and reported [108]. In 1993, research teams employed gene cloning techniques to isolate the *HTT* gene and utilized methods such as Southern blotting and PCR to uncover the pathogenic cause of HD [10,37,38]. They discovered that the abnormal elongation of the CAG repeat sequence in exon 1 of the *HTT* gene results in the formation of an excessively long polyQ structure at the N-terminus of the huntingtin protein [109,110]. This mutant protein adopts misfolded conformations within neurons and has a propensity to aggregate, forming inclusions, thereby impairing neuronal function and ultimately contributing to the onset and progression of the disease [111,112]. Similarly, in 1922, researchers employed STR linkage analysis in conjunction with trinucleotide repeat-primed PCR (TP-PCR) technology to identify the presence of CTG repeat expansions in the 3′ UTR of the *DMPK* gene in patients with type 1 myotonic dystrophy (DM1). The expanded CTG repeat sequences are transcribed into CUG repeat RNA, which forms hairpin structures that aberrantly bind RNA-binding proteins (RBPs), such as muscleblind-like splicing regulator 1 (MBNL1). This interaction disrupts splicing regulation, leading to multisystem dysfunction [113,114]. In addition to the aforementioned disorders, numerous other repeat expansion disorders fall under the category of single-gene inheritance patterns [32,33].

With the rapid advancement of sequencing technology and the widespread application of large-scale sequencing in the study of STRs [24,25,26], numerous studies have shown that many STR loci are associated with multifactorial complex diseases [28,29,30,49,115,116,117]. Currently, modern STR variant detection technology integrates traditional detection methods (such as PCR detection and Southern blot analysis) [58,59], various sequencing techniques (including NGS and LRS), and a variety of STR genotyping tools [3,31]. This integration has achieved the precise diagnosis and treatment of multifactorial complex diseases, providing strong support for the precise diagnosis and treatment of such diseases. Similarly, a 2025 study on Alzheimer’s disease (AD) utilized whole-genome sequencing (WGS) on AD patients and control groups, integrating bioinformatics tools such as ExpansionHunter for the comprehensive detection of STR expansion loci, lengths, and frequency distributions [97]. The study found that AD patients exhibited a 1.19-fold increase in STR expansions compared to healthy elderly controls. Individuals with over 30 STR expansions had a 3.69-fold increased risk of developing AD and presented with more severe neuropathological features of the disease [97]. Further analysis revealed the significant enrichment of AD-associated STR expansions in active promoter regions of postmortem hippocampal tissue, particularly within SINE-VNTR-Alu (SVA) retrotransposons [97]. This suggests a tight correlation between STR expansions in active promoter regions and AD risk [97]. The ExpansionHunter demonstrated high accuracy in estimating STR numbers and reliably identified STRs with normal lengths under 150 base pairs as well as abnormal expansions exceeding 200 base pairs, proving its efficacy in handling long repeat sequences and complex structural variations [95,118,119].

## 4. The Genetic Characteristics of Mental Disorders Associated with Tandem Repeats

STRs have long been regarded as “genetic stutters” with little functional relevance [120,121]. However, mounting evidence now implicates these dynamic mutations as key players in the pathogenesis of Mental Disorders [20,122]. Unlike single nucleotide variants, STRs exert their influence through repeat expansions or contractions that disrupt gene expression, protein function, and neural circuitry [30,123,124]. However, the role of these tandem repeats in polygenic Mental Disorders remains largely elusive [28,125]. Even so, over the past few decades, researchers have gained new insights into the psychiatric disorders caused by TREs, thanks to the continuous advancements in technological methodologies.

In a 2020 study, researchers harnessed WGSdata from the MSSNG project [126], the Simons Simplex Collection (SSC) [127], and the 1000 Genomes Project [128] to meticulously explore the role of TREs in ASD [29]. Utilizing the EHdn algorithm [75] alongside a suite of validation techniques such as PCR, Sanger sequencing, RP-PCR, and Southern blot, they conducted an in-depth analysis [29]. The findings revealed that rare TREs are significantly more prevalent in individuals with ASD compared to their unaffected siblings [29]. This prevalence was notably higher in regions proximal to exons and splice junctions, particularly within genes implicated in neurodevelopment and the cardiovascular or muscular systems [29]. Collectively, these rare TREs account for a 2.6% contribution to ASD risk. Among the genes exhibiting such expansions are *DMPK* and *FXN*, as well as *FGF14* and *CACNB1* [29].

Another 2021 study conducted an in-depth analysis of WGS data from the SSC [127], utilizing advanced algorithms such as GangSTR [102] and MonSTR [28]. The study uncovered that the genomes of children with ASD harbored a significantly higher number of TR mutations, which were also larger in scale, compared to their unaffected siblings [28]. These mutations were predominantly concentrated in regulatory regions of genes tied to fetal brain development, suggesting a potential role in modulating gene expression and thus influencing neurodevelopmental processes [28]. Furthermore, from an evolutionary perspective, these mutations appear more likely to be deleterious, potentially affecting the pathogenesis of ASD through alterations in protein function or regulatory elements [28,129]. Notably, new TR mutations arising from the maternal germline were found to be larger in size than those originating from the paternal germline and were more prone to instigate gradual changes in copy number [28].

Recent research focusing on the expansion of STRs in cortex-specific genes has meticulously analyzed WGS data from 634 ASD families [53]. Utilizing the STRling algorithm [76] alongside various validation techniques such as satellite marker analysis, RP-PCR, and nanopore LRS, the study provided profound insights [53]. It was discovered that rare STR expansions predominantly occur in early cortical development genes involved in neurodevelopment within the ASD patient population [53]. This expansion disrupts the regulatory activity of enhancers and promoters, thereby contributing to the pathogenesis of ASD [53]. Furthermore, individuals carrying ASD-associated STR expansions exhibit more pronounced ASD phenotypic characteristics and experience reduced adaptive abilities compared to non-carriers [53].

Another study on SCZ conducted WGS on 1154 Swedish patients diagnosed with SCZ and 934 matched controls [130]. The findings revealed a significantly higher burden of rare TREs in SCZ patients, particularly within genic regions, such as postsynaptic genes, brain-expressed genes, and genes that are differentially expressed between SCZ patients and controls. Furthermore, these TRE-associated genes are often more constrained, primarily affecting synaptic and neuronal signaling functions.

In a 2018 study, researchers analyzed 181 human cell lines (encompassing diverse ethnic groups) and postmortem brain tissue samples, with a particular focus on neural tissue [131]. This investigation combined LRS technologies, such as PacBio, with GWAS to elucidate the tandem repeat variations in the third intronic region of the *CACNA1C* gene, aiming to validate the polymorphism of STRs and their associations with BD and SCZ. The results revealed that the tandem repeat located within the non-coding region of the third intron of the *CACNA1C* gene consists of a 30 bp unit repeated hundreds of times, with lengths reaching several kilobases [131]. This repeat exhibits enhancer activity, capable of activating reporter gene expression in human neural progenitor cells. Different allelic variants of the repeat array displayed varying enhancing activities, with risk-associated alleles demonstrating significantly reduced enhancer functionality [131]. The aberrant expression linked to these alleles may influence neuronal excitability and synaptic plasticity through the calcium signaling pathway, thereby increasing the susceptibility to psychiatric disorders [131].

Furthermore, STR variations are associated with a multitude of other psychiatric disorders. For instance, in Attention Deficit Hyperactivity Disorder (ADHD), the variable number tandem repeats (VNTRs) in the *DAT1* gene, particularly the 10-repeat allele, have been linked to an increased risk of ADHD, potentially influencing the efficiency of dopamine reuptake [132].

For another instance, in the context of depression, certain tandem repeat variations within the *BDNF* gene may contribute to the pathogenesis by influencing the expression of neurotrophic factors. However, the precise mechanisms underlying this association require further validation [133].

Overall, these studies suggest that short TREs may play a pivotal role in Mental Disorders (Table 2), necessitating further functional studies to elucidate the specific mechanisms of STR involvement [134].

## 5. The Genetic Characteristics of Repeat Expansion Disorders

As research delves deeper into the significance of STRs, coupled with advancements in sequencing technologies, scientists have identified several common genetic characteristics inherent in STR expansion disorders [25,77,118]. A notable aspect of these disorders is that they rarely present as a simple binary contrast between healthy and affected individuals. Instead, they manifest a spectrum of quantifiable traits, such as the age of onset and the severity of symptoms, which are modulated by the number of tandem repeats [49]. Due to the intergenerational instability of these tandem repeat sequences, they are regarded as dynamic mutations [135]. Variations in repeat number within certain ranges can lead to differences in age of onset, severity, penetrance, and clinical phenotype [136,137,138]. Thus, dynamic mutations can be categorized into full mutations, pre-mutations, gray zones, and normal alleles [9,139].

Full mutation alleles are pathogenic and exhibit somatic instability, often increasing in size when passed to subsequent generations [20,140]. In FXS, individuals with over 200 CGG repeats in the *FMR1* gene’s promoter region experience hypermethylation and transcriptional silencing, leading to a deficiency in fragile X mental retardation protein (FMRP), crucial for CNS synaptic plasticity [35,36]. Similarly, over 1000 CTG repeats in the *DMPK* gene cause congenital DM1 [141]. These characteristics influence the age of onset, progression, and severity of certain repeat expansion disorders [142,143].

Pre-mutation alleles, positioned between normal and fully mutated states, may result in mild or variable phenotypes [144,145,146]. For instance, pre-mutations like 50–150 CTG repeats in the *DMPK* gene are associated with cataracts and mild myotonia, whereas higher repeat counts, such as 100–1000 CTG repeats, lead to classic severe conditions like muscle weakness and cardiac arrhythmias [147,148]. Furthermore, pre-mutations are characterized by genetic instability, with a pronounced tendency to expand into full mutations during maternal transmission. And pre-mutations could enhance promoter activity and transcription, leading to excessive RNA production and the formation of toxic aggregates, which directly impair cellular function [49,149].

The gray zone encompasses repeat sequences that lie between normal and pathogenic ranges, typically not causing disease but potentially expanding in subsequent generations [150,151]. Normal repeat sequences are vital for biological functions, regulating gene expression by influencing promoter activity [152,153].

Another genetic characteristic of STR expansion disorders is that their clinical manifestations and pathogenic mechanisms are influenced by the composition of the STR motifs. For instance, expanded alleles composed of uninterrupted repeat motifs often exhibit a high degree of structural instability [54,135]. For example, the abnormal expansion of the hexanucleotide repeat GGGGCC in the *C9orf72* gene can form secondary structures such as G-quadruplexes [154,155]. These structures can disrupt DNA replication and repair processes, leading to replication fork stalling or slippage, which in turn exacerbates repeat expansion or contraction [155,156,157]. This mechanism is implicated in the pathogenesis of amyotrophic lateral sclerosis (ALS) and frontotemporal dementia (FTD) [158]. Notably, the genetic stability of certain STR loci can be significantly enhanced through the interruption of their repeat sequences by heterologous motifs. A classic example is found in the *FMR1* gene associated with FXS. The insertion of AGG trinucleotides at regular intervals within the CGG repeat sequence effectively suppresses the conversion of pre-mutation alleles (55–200 repeats) to full mutation states (>200 repeats) during maternal transmission, substantially reducing the risk of FXS in offspring [145,159,160,161]. Moreover, there is a notable correlation between specific motif types and disease phenotypes. For instance, CGG-rich repeat sequences, due to their propensity to form unique secondary structures, often disrupt the expression regulation of genes involved in neurodevelopment, thereby showing significant pathogenic associations with various neurodevelopmental disorders, such as FXS and FXTAS [162].

In the investigation of genetic diseases, the disparities in STR variations among different ethnicities or specific populations, as well as their associations with neuropsychiatric disorders, may be closely linked to the founder effect [20]. Notably, larger repeat expansions are often associated with single or predominant haplotypes, resulting in the clustering of diseases within particular geographic regions or leading to significant variations in prevalence rates [20]. For instance, DM1 and Friedreich’s ataxia (FRDA) are exceedingly rare in Africa and Japan [163,164], suggesting that non-expanded alleles remain relatively stable, while risk haplotypes exist in a state of linkage disequilibrium with large normal alleles at homologous repeat loci [158]. This phenomenon implies a potentially multistep evolutionary process, where an initial mutation gives rise to a large normal allele, which subsequently expands over time, ultimately resulting in pathogenic alleles [20]. In a study conducted in 2023, researchers analyzed the STR length variability in 850 children suspected of having genetic disorders and 996 parents, encompassing over a thousand loci from six distinct ancestral groups (Africans, Europeans, East Asians, admixed Americans, non-admixed Americans, and Pacific Islanders) [165]. The results revealed that the STR lengths in admixed Americans were most similar to those of Europeans, while the African population exhibited the greatest variability. The study also identified significant expansions of STRs in known pathogenic genes such as *TCF4, AR,* and *DMPK* across multiple individuals, with lengths exceeding established pathogenic thresholds [165]. Additionally, it confirmed individuals with significant expansions at highly conserved loci and discovered two cases of exonic STR expansions associated with X-linked intellectual disability [165]. Overall, this research lays a foundation for understanding the differences in STR variations across different ethnicities or specific populations, providing crucial insights into the underlying mechanisms of certain genetic disorders.

## 6. The Pathogenic Mechanisms of STRs

Repeat expansion disorders have diverse pathogenic mechanisms, which vary depending on the location of the expanded STR within the gene locus. STRs can occur in exons, introns, untranslated regions, or intergenic areas [30]. These region-specific STRs can interfere with one another through diverse pathogenic mechanisms, ultimately collaborating to culminate in the manifestation of disease [21]. Current research generally suggests that repeat expansion disorders cause disease through the following four molecular mechanisms [21,162].

Loss of gene function is the inhibition of gene transcription caused by nucleotide repeat amplification, which affects normal gene expression and function. For example, FXS is caused by a CGG repeat amplification on the X chromosome, which results in methylation and silencing of the *FMR1* gene [35,166]. This results in the absence of the FMRP protein, which affects neurodevelopment and synaptic plasticity, ultimately leading to disease onset [167].

RNA-mediated gain of function is associated with the formation of aberrant structures, such as hairpin structures or circular RNA, in RNA molecules produced by nucleotide repeat amplification [45,168,169]. These structures interact with RBPs, leading to the disruption of RNA metabolism and transcriptional regulation. In ankylosing muscular dystrophy disease, the amplification of CTG repeats in the *DMPK* gene results in abnormal RNA molecules containing CUG repeat sequences [170,171]. These molecules bind to the MBNL1 protein and form nuclear inclusions that affect splicing regulation and muscle function leading to disease [45,46,47,172].

The gain of function of classic translation proteins containing expanded repeat sequences occurs when nucleotide repeat amplification takes place within the coding region of a gene, resulting in the production of proteins with abnormal amino acid repeat sequences, which could lead to increased toxicity or altered protein function [18,173]. For example, HD is caused by the amplification of CAG repeats in the *HTT* gene, which leads to the production of proteins with polyQ sequences in the gene. This causes protein aggregation and the disruption of neuronal survival and signaling, which ultimately leads to disease [9,38].

Repeat-associated RAN translation refers to a non-canonical translation process using non-AUG start codons to translate RNA molecules containing expanded nucleotide repeat sequences. This process results in polypeptide chains with abnormal amino acid repeats that form inclusions within cells. For instance, in ALS and FTD, the expansion of G4C2 repeat sequences leads to the production of polypeptides like polyG and polyP through RAN translation [174]. These polypeptides aggregate into cytotoxic inclusions that disrupt protein homeostasis, organelle function, and cell signaling, potentially damaging cell membranes and leading to cell death, particularly in neurons [169,175,176]. In diseases such as ALS and FTD, the accumulation of these peptides in neurons significantly contributes to neuronal dysfunction and disease progression [169,176,177,178,179].

Research has demonstrated that the synergistic effects of diverse pathogenic mechanisms may contribute to the onset of these disorders, with multifactorial pathological mechanisms being a prevalent phenomenon rather than an exception in neuropsychiatric diseases [21]. For instance, in C9 ALS/FTD, the abnormal expansion of the GGGGCC hexanucleotide repeat within the intronic region of the *C9orf72* gene represents a major genetic etiology [180]. This expanded repeat sequence induces intron retention, resulting in diminished expression of the *C9orf72* gene, while concurrently extending the half-life of the repeat sequence RNA, thereby exacerbating the sequestration of RBPs [181,182,183]. Furthermore, intron retention may also enhance the production of dipeptide repeat sequences, activating multiple downstream pathogenic pathways [180]. In HD, neurotoxicity is instigated through a dual mechanism wherein the expanded CAG repeat sequence alters RNA processing, affecting the recognition of the donor splice site for exon 1 [184]. This alteration leads to the production of a truncated HTT protein characterized by a polyQ) stretch, a form of the protein that exhibits heightened toxicity [185]. Additionally, RAN translation may also occur within the CAG repeat sequence. In FXTAS/FXS, the CGG repeat sequence not only has the capacity to sequester RBPs [186] but also enhances the RAN translation of upstream open reading frames (uORFs), resulting in a reduced initiation of FMRP translation [167,187].

## 7. The Common Animal Models Used in the Study of STR Variations in Neuropsychiatric Disorders

In the realm of psychiatric research, the utilization of animal models is of paramount importance. Each type of animal model possesses unique characteristics that allow for the effective simulation of human psychiatric disorders through the application of CRISPR/Cas9 technology to knock out or insert specific STR loci.

Rodents, such as mice and rats, are widely employed in studies to model diseases like SCZ, ASD, and depression [95]. For instance, the YAC128 mouse model for HD expresses the full-length human *HTT* gene with an expanded CAG repeat sequence [188]; conditional frataxin knock-out mouse models involve the deletion of the *FXN* gene [189]; and knock-in mouse models for fragile X-associated tremor/ataxia syndrome (FXTAS) feature a CGG repeat insertion [190]. The advantages of utilizing rodent models include the relative ease of constructing STR mutation models. Additionally, these animals are cost-effective, breed rapidly, and are suitable for the large-scale screening of drug effects or genetic interventions [191]. However, notable limitations exist. Rodent brains exhibit insufficient neuroanatomical complexity; their prefrontal cortex and other advanced brain regions are underdeveloped, making it challenging to fully replicate human psychiatric diseases [192]. Furthermore, the phenotypic expression in these models is limited, as certain symptoms—such as language deficits and complex cognitive impairments—cannot be adequately represented through animal behavior [193,194].

In contrast, non-human primates, such as macaques and marmosets, offer unique advantages for investigating the impact of STR variations on social behavior and cognitive function. For example, macaque models can be employed to explore the association between STR polymorphisms in the *AVPR1a* gene and social behaviors [195]. The benefits of using non-human primates include their highly conserved neuroanatomical structures and functions, with the prefrontal cortex and limbic system exhibiting remarkable similarity to those of humans, making them particularly suitable for exploring complex cognitive disorders [196,197]. Additionally, they can model intricate behaviors, such as social hierarchies and cooperative interactions [196,197]. However, significant drawbacks are also evident. The costs associated with using non-human primates can be prohibitively high, and their developmental timelines are long, with sexual maturity occurring at four to five years of age [198]. Furthermore, the expenses related to gene editing in these animals are substantial [198].

Zebrafish are primarily employed to study the effects of STR variations on early neurodevelopment, exemplified by ASD-like behaviors associated with mutations in the shank3b gene [199]. The advantages of using zebrafish include their suitability for high-throughput screening; and their transparent embryos facilitate in vivo imaging and rapid genetic screening [200]. Additionally, they are cost-effective and have a short lifecycle, with embryonic development completing within five days, making them ideal for large-scale drug testing [200]. However, considerable limitations are present. The behavioral phenotypes exhibited by zebrafish are relatively simplistic and fail to capture complex cognitive symptoms, such as deficits in working memory [201]. Moreover, the neural circuits in zebrafish differ from those in mammals, and their lack of cortical structures restricts the depth of mechanistic studies [201].

## 8. Conclusions

In conclusion, STRs, once considered inconsequential “junk DNA,” are now recognized as critical players in the complex landscape of neuropsychiatric disorders. This review has highlighted the multifaceted nature of STR pathogenicity, ranging from direct gene disruption through repeat expansions leading to loss of function or toxic gain of function (via RNA, protein, or RAN translation mechanisms) to more subtle modulations of gene expression impacting neurodevelopment and neuronal signaling. Advanced sequencing technologies, particularly LRS, are revolutionizing our ability to detect and characterize STR variations, revealing a spectrum of repeat instability and its correlation with disease onset, severity, and phenotypic expression. Compelling evidence links STR expansions to a growing list of neuropsychiatric conditions, including ASD and SCZ, solidifying their contribution to disease risk. While considerable progress has been made, further research is crucial to fully unravel the intricate interplay between STRs and these complex disorders. This includes investigating the functional consequences of specific STR expansions, exploring potential therapeutic targets based on the identified pathogenic mechanisms, and developing more precise diagnostic tools for improved patient stratification and personalized interventions. The ongoing exploration of STRs promises to unlock new avenues for understanding and ultimately treating these debilitating conditions.

## Figures and Tables

**Table 1:** Comprehensive table of neuropsychiatric disorder-associated STR variants and inheritance patterns.

Gene	Diseases	OMIM ID	Repeat Motif	Mode of Inheritance	Location on Gene	Mechanism	Age of Onset	Repeat Arrangement in GRCh38
*HTT*	HD	143100	CAG	AD	Exon 1	GoF (polyQ) GoF (RNA) RAN translation	Pediatric/Adult (Mean: 35–44)	(CAG)19 CAA CAG CCG CCA (CCG)7
*FMR1*	FXTAS/ FXPOI	300623	CGG	XLD	5′ UTR	GoF (RNA) RAN translation	Adult (FXTAS: 60–65. FXPOI < 40)19	(CGG)10 AGG (CGG)9
*FMR1*	FXAND	-	CGG	XLD	5′ UTR	GoF (RNA) RAN translation	Pediatric/Adult	(CGG)10 AGG (CGG)9
*FMR1*	FXS	300624	CGG	XLD	5′ UTR	LoF	Pediatric	(CGG)10 AGG (CGG)9
*C9ORF72*	FTD and/ or ALS1	105550	GGGGCC	AD	Intron 1	LoF GoF (RNA) RAN translation	Adult (Typical: 50–64)	(GGGGCC)3
*ATXN1*	SCA1	164400	CAG	AD	Exon 8	GoF (polyQ) LoF	Adult (Typical: 30–40)	(CAG)12 CAT CAG CAT (CAG)14
*ATXN2*	SCA2/ ALS13	183090	CAG	AD	Exon 1	GoF (polyQ) GoF (RNA) RAN translation	Adult (Typical: 40)	(CAG)13 CAA (CAG)9
*ATXN3*	SCA3/ MJD	109150	CAG	AD	Exon 10	GoF (polyQ) GoF (RNA) RAN translation	Pediatric/Adult (Typical: 20–50)	(CAG)2 CAA AAG CAG CAA (CAG)8
*ATXN7*	SCA7	164500	CAG	AD	Exon 3	GoF (polyQ) GoF (RNA)	Pediatric/Adult (Typical: >40)	(CAG)10
*ATXN8OS/* *ATXN8*	SCA8	608768	CTG/CAG	AD	3′ UTR exon 5	GoF (polyQ) GoF (RNA) RAN translation	Pediatric/Adult (Typical: 30–50)	(CTG/CAG)15
*ATXN10*	SCA10	603516	ATTCT	AD	Intron 9	GoF (RNA)	Pediatric/Adult (Range: 12–48)	(ATTCT)14
*TCF4*	FECD3	613267	CTG	AD	Intron 3	GoF (RNA) RAN translation	Adult	(CTG)24
*PRNP*	CJD	123400	CCTCATGGTGGTGGCTGGGGGCAG	AD	Exon 2	LoF?	Adult	(CCTCATGGTGGTGGCTGGGGGCAG)2 CCCCATGGTGGTGGCTGGGGGCAG CCTCATGGTGGTGGCTGGGGTCAA
*ARX_1*	DEE1/ XLID29	308350/300419	NGC	XLR	Exon 2	LoF	Pediatric	(NGC)16
*ARX_2*	DEE1/ PRTS/ XLID29	308350/309510/300419	NGC	XLR	Exon 2	LoF	Pediatric	(NGC)12
*TBP*	SCA17	607136	CAG	AD	Exon 3	LoF GoF (polyQ)	Pediatric/Adult (Mean: 34.6)	(CAG)3 CAA CAA CAA (CAG)8 CAA CAG CAA (CAG)19
*ZIC2*	HPE5	609637	GCN	AD	Exon 3	LoF	Pediatric	(GCN)15
*ZIC3*	VACTERLX	314390	GCC	XLR	Exon 1	Unknown	Pediatric	(GCC)8 GCT GCC
*NOTCH2NLC*	NIID/ETM6/PD	603472/618866/618600	GGC	AD	5′ UTR	GoF (RNA) GoF (polyG)	Adult (Mean: 60.5)	(GGC)9 GGA GGA(GGC)2
*DMPK*	DM1	160900	CTG	AD	3′ UTR	GoF (RNA) RAN translation	Pediatric/Adult (Range: birth–70)	(CTG)20

HD, Huntington’s disease; FXTAS, fragile X-associated tremor/ataxia syndrome; FXPOI, fragile X-associated primary ovarian insufficiency; FXAND, fragile X-associated neuropsychiatric disorders; FXS, fragile X syndrome; FTD, frontotemporal dementia; ALS, amyotrophic lateral sclerosis; SCA, spinocerebellar ataxia; MJD, Machado–Joseph disease; FECD, Fuchs endothelial corneal dystrophy; CJD, Creutzfeldt–Jakob disease; DBQD, Desbuquois dysplasia; DEE, developmental and epileptic encephalopathy; XLID, X-linked intellectual developmental disorder; HPE, holoprosencephaly; VACTERLX, X-linked VACTERL association with or without hydrocephalus; NIID, neuronal intranuclear inclusion disease; ETM, hereditary essential tremor; PD, Parkinson’s disease; DM, myotonic dystrophy; GoF, gain of function; RAN, repeat-associated non-AUG; LoF, loss of function.

**Table 2:** Main findings and characteristics of STRs in mental disorders associated with tandem repeats.

Disease	Associated Genes/Locations	Functional Impact	Genetic Characteristics	Key Findings	Year of Study
ASD	*DMPK*, *FXN*, *FGF14*, *CACNB1*, genes related to cortical development	Abnormalities in neurodevelopment, cardiovascular/muscular system gene functions; disruption of regulatory regions (enhancer/promoter)	Maternal mutations tend to be more substantial; higher frequency of new TR mutations (compared to unaffected siblings)	Rare tandem repeats (TREs) contribute to 2.6% of ASD risk Mutations are enriched near exons/splice sites and regulatory regions of fetal brain development Carriers of STR expansions exhibit more severe phenotypes and lower adaptive capacity	2020, 2021, 2023
Schizophrenia	Postsynaptic genes, brain-expressed genes (e.g., intronic region of *CACNA1C*)	Abnormal synaptic signaling, neuronal excitability, and calcium signaling pathways	Higher burden of rare TREs in patients; reduced enhancer activity of risk alleles	TREs are enriched in gene regions (e.g., postsynaptic genes) Length of intronic STR in *CACNA1C* influences enhancer activity, correlating with disease risk	2021, 2023
Bipolar Disorder	Non-coding region of the third intron of *CACNA1C* gene (30 bp repeat unit)	Variations in enhancer activity lead to abnormalities in neuronal excitability and synaptic plasticity	Significantly reduced enhancer activity of risk alleles	Long STR alleles are associated with increased risk of BD/SCZ Repeat lengths can reach thousands of base pairs, impacting calcium signaling pathways	2018
Attention Deficit Hyperactivity Disorder (ADHD)	VNTR of the *DAT1* gene (10-repeat allele)	Altered dopamine reuptake efficiency	Variations in dopamine transporter-encoding gene	The 10-repeat allele is associated with an increased risk of ADHD	2015
Depression	Tandem repeats in the *BDNF* gene	Abnormal expression of neurotrophic factors	Mechanism not fully understood	Specific STR variations may contribute to pathogenesis by influencing *BDNF* expression	2009

## Data Availability

The data for this review are sourced from publicly available literature, primarily through comprehensive literature searches conducted in databases such as PubMed.
